# Production and characterisation of a marine *Halomonas* surface-active exopolymer

**DOI:** 10.1007/s00253-019-10270-x

**Published:** 2019-12-07

**Authors:** Tony Gutierrez, Gordon Morris, Dave Ellis, Barbara Mulloy, Michael D. Aitken

**Affiliations:** 1 Institute of Mechanical, Process and Energy Engineering (IMPEE), School of Engineering and Physical Sciences, Heriot-Watt University, Edinburgh, UK; 2 Department of Environmental Sciences and Engineering, Gillings School of Global Public Health,, University of North Carolina, Chapel Hill, NC USA; 3 Department of Chemical Sciences, School of Applied Sciences, University of Huddersfield, Huddersfield, UK; 4 Institute of Chemical Sciences (ICS), School of Engineering and Physical Sciences, Heriot-Watt University, Edinburgh, UK; 5 Institute of Pharmaceutical Science, King’s College London, London, UK

**Keywords:** Exopolymers, *Halomonas*, Hydrocarbons, Biodegradation, Marine environment

## Abstract

During screening for novel emulsifiers and surfactants, a marine gammaproteobacterium, *Halomonas* sp. MCTG39a, was isolated and selected for its production of an extracellular emulsifying agent, P39a. This polymer was produced by the new isolate during growth in a modified Zobell’s 2216 medium amended with 1% glucose, and was extractable by cold ethanol precipitation. Chemical, chromatographic and nuclear magnetic resonance spectroscopic analysis confirmed P39a to be a high-molecular-weight (~ 261,000 g/mol) glycoprotein composed of carbohydrate (17.2%) and protein (36.4%). The polymer exhibited high emulsifying activities against a range of oil substrates that included straight-chain aliphatics, mono- and alkyl- aromatics and cycloparaffins. In general, higher emulsification values were measured under low (0.1 M PBS) compared to high (synthetic seawater) ionic strength conditions, indicating that low ionic strength is more favourable for emulsification by the P39a polymer. However, as observed with other bacterial emulsifying agents, the polymer emulsified some aromatic hydrocarbon species, as well as refined and crude oils, more effectively under high ionic strength conditions, which we posit could be due to steric adsorption to these substrates as may be conferred by the protein fraction of the polymer. Furthermore, the polymer effected a positive influence on the degradation of phenanthrene by other marine bacteria, such as the specialist PAH-degrader *Polycyclovorans algicola*. Collectively, based on the ability of this *Halomonas* high-molecular-weight glycoprotein to emulsify a range of pure hydrocarbon species, as well as refined and crude oils, it shows promise for the bioremediation of contaminated sites.

## Introduction

Surface-active agents are a group of chemicals that play an important role in various industrial processes and products due to their ability to interface between hydrophobic (non-aqueous) and hydrophilic (aqueous) phases. These chemicals can be divided into two major types: (1) Surfactants, which are generally of low-molecular-weight and denoted by their ability to reduce surface and/or interfacial tension between two-phase media, and (2) emulsifiers, which are biopolymers of high-molecular-weight and are characterised by their ability to form oil-in-water or water-in-oil emulsions and/or in stabilizing the emulsion droplets. Surfactants and emulsifiers are used in almost every sector of modern industry, particularly in personal care products and household cleaners. To fulfill their demand, the majority of these chemicals are produced from non-renewable sources, such as petrochemicals. In recent years, however, efforts to discover new types of these chemicals from natural sources—denoted as biosurfactants and bioemulsifiers—have gained pace. Surface-active agents produced from natural sources are environmentally friendlier compared to their synthetic counterparts, as their biosynthesis can be performed using renewable feedstocks (e.g. agricultural waste) and importantly they are biodegradable (Panilaitis et al. 2006).

The marine environment is considered a largely untapped source for the discovery of new types of surface-active agents. This is highlighted in the enormous diversity of bacteria that exists in the world’s seas and oceans that could yield novel types of these biomolecules (Kalogerakis et al. 2015). Marine bacteria can secrete exopolymeric substances (EPS) that can consist of any combination of proteins and/or lipids within a major polysaccharide backbone. These macromolecules serve important functions to the producing microorganism(s) and its neighbours, as well as to animals and plants, and also have wider implications to the marine environment, including to global biogeochemical cycles and climate change (Decho and Gutierrez 2017). For example, EPS produced by marine bacteria have been shown involved in sequestering dissolved cationic species (Loaec et al. 1997, 1998; Steiner et al. 1976) and, more broadly, in biogeochemical processes (see Decho and Gutierrez 2017). These macromolecules have also been shown to mediate the initial attachment of bacteria to surfaces in biofilm formation (Decho 2000a, b; Thavasi and Banat 2014), in interacting with dissolved and/or particulate organic matter (Decho and Gutierrez 2017 and references therein), including in the formation of marine oil snow (Gutierrez et al. 2013b) and aggregates with micro- and nano-plastic particles in seawater (Summers et al. 2018).

Halomonads are slight to moderately halophilic and oligotrophic organisms that are ubiquitous to marine and hypersaline environments (Arahal and Ventosa 2006) and recognised for producing large quantities of EPS. EPS from various *Halomonas* species have been reported to confer excellent rheological properties (Arias et al. 2003; Bèjar et al. 1996; Bouchotroch et al. 2000; Calvo et al. 1998; Martinez-Checa et al. 2002; Mata et al. 2006) and to bind transition metals and dyes (Arias et al. 2003; Gutierrez et al. 2009; Mata et al. 2006). Some species have been reported to produce EPS with surface-active properties, which may provide a mechanism to scavenge poorly soluble, hydrophobic substrates (e.g. lipids, hydrocarbons) that the cells can then utilise for growth in the marine environment. For example, the EPS of some *Halomonas* spp. has been shown to increase the bioavailability of aliphatic (Pepi et al. 2005) and aromatic (Alva and Peyton 2003; Garcia et al. 2004, 2005; Gutierrez et al. 2013b) compounds. In this study, we characterise the EPS produced by a *Halomonas* species that was isolated from a coastal surface seawater environment. We investigate its production during growth of the organism in a batch fermenter, and analyse the chemico-physiological properties of the extracted EPS, including its potential to emulsify a range of crude and refined petrochemical and hydrocarbon substrates. Based on our findings, we offer potential avenues to develop the biopolymer for biotechnological application, including as a promising candidate for the bioremediation of sites contaminated with petrochemical pollutants.

## Materials and methods

### Strain isolation, growth and identification

Surface water samples were collected in July of 2009 off Long Beach, California. To isolate hydrocarbon-degrading bacteria, a synthetic seawater medium, ONR7a (Dyksterhouse et al. 1995), was supplemented with *n*-hexadecane (Hex) to 1% (v/v) final concentration. Isolation was performed by streaking 5 μl samples of the seawater onto the ONR7a+Hex agar plates. The plates were stored in closed plastic bags in the dark at 21 °C for 2 to 3 weeks. Isolates displaying distinct colonial morphologies were picked, purified and examined for growth on *n*-hexadecane as the sole carbon and energy source. Purified isolates were stored frozen at − 80 °C in 20% (v/v) glycerol for subsequent sequencing and further work.

One isolate, strain MCTG39a, was selected for its ability to produce high emulsifying activity during growth in ZM/1 broth (Blackburn et al. 1989) amended with glucose at 1% (w/v) concentration. During growth, samples were taken periodically for emulsification assays—washed cell pellets and cell-free supernatants (centrifuged at 13,000×*g* for 10 min) were assayed. Growth was monitored by spectrophotometric measurement at 600 nm of whole culture broth. Growth experiments were repeated at least three times, and all analyses were performed in triplicate.

Total genomic DNA from MCTG39a was recovered using a Wizard genomic DNA purification kit (Promega, Madison, WI), according to the manufacturer’s instructions. The 16S rRNA gene of the strain was amplified by PCR with primers 27f (Wilmotte et al. 1993) and 1492r (Lane 1991), and then sequenced at the University of North Carolina Genome Analysis Facility. Sequences were analysed using the program Sequencher 4.8 (Gene Codes Corp., Ann Arbor, MI) and submitted to GenBank. The BLAST search program and RDP-II (Maidak et al. 1999) were used to check for close relatives and phylogenetic affiliation. The search results were used as a guide for tree construction. Clustal_X (Thompson et al. 1994) was used to align the 16S rRNA gene sequence of strain MCTG39a with close relatives and to construct a neighbour-joining tree with Treeview (WIN32) version 1.5.2 (Page 1996). The trees were bootstrapped 1000 times, and gaps in the alignment were ignored.

### Production and extraction of emulsifying exopolymer

Strain MCTG39a was grown in a 14-L bench-top FerMac 320 (Electrolab, UK) fermenter containing 4 l of ZM/1 medium amended with glucose (1% w/v) and incubated at 28 °C with mixing at 150 rev min^−1^. The fermenter was fitted with temperature, pH and dissolved oxygen probes (Broadley James, USA), and data was captured with FermaTec software (Electrolab, UK). To monitor production of the emulsifying exopolymer, samples (2 mL) from the fermenter were taken daily and the emulsifying activity of the cell-free spent medium assayed.

The fermenter was terminated when maximal emulsifying activities were reached (EI_24_ > 100%). The emulsifying exopolymer was then extracted from the biomass by centrifugation (10,000×*g*; 20 min) of the spent medium followed by filtration through 0.2 μm filters. To isolate the emulsifying exopolymer, the cell-free permeate was treated with KCl (7.5% w/v) and two volumes of cold 99% ethanol. The precipitate was allowed to settle overnight at 4 °C, recovered by centrifugation, and subsequently dialysed against distilled water and freeze-dried. The resultant dried material, labeled P39a, was used in all subsequent characterization experiments.

### Emulsification assays and tensiometry

To assay for emulsifying activity during growth of strain MCTG39a, a modified version of the method described by Cooper and Goldenberg (1987) was used. For this, samples of culture broth were centrifuged (13,000×*g*; 10 min) and then filtered (0.2 μm) to completely remove cells. For the emulsification assay, 2 ml of cell-free fraction was mixed with an equal volume of *n*-hexadecane in acid-washed (0.1 N HCl) screw-cap glass tubes (100 × 13 mm). The tubes were then manually shaken (15 s), vigorously vortexed (15 s) to homogeneity, left to stand for 10 min and then shaken as before. After allowing the mixture to stand for 24 h at 21 °C, the height of the emulsion layer was measured and expressed as a percentage (EI_24_) of the total original height of the oil in the tube.

To determine the specificity of the P39a exopolymer to emulsify various hydrocarbon substrates, including some crude oils and their refined petro-derivatives, we employed a modified emulsification assay based on that described by Cirigliano and Carman (1984). For this, 2.5-ml solutions of the extracted polymer (0.02% w/v) dissolved in 0.1 M phosphate-buffered saline (PBS; pH 7.0) or filtered (0.2 μm) synthetic filtered seawater (FSW; pH 8.0) were emulsified against 0.4 ml of the test oil, as described previously (Gutierrez et al. 2007). After mixing, the solutions were allowed to stand for 60 min prior to measuring the absorbance of the lower aqueous phase by spectrophotometry at 540 nm. Emulsification activities are expressed as the average absorbance at 540 nm values derived from triplicate experiments when testing each of the oils in PBS or FSW.

Surface tension measurements were performed using a Kibron EZ-Pi Plus surface tensiometer equipped with a Dyne probe for static du Noüy ring measurements. Samples of strain MCTG39a growing in ONR7a or ZM/1 broth, and of cell-free fractions, were measured for surfactancy. The isolated P39a exopolymer at various concentrations dissolved in water were also measured to evaluate its potential to reduce the surface tension of water.

### Chemical analysis of the exopolymer

To determine the monosaccharide composition, triplicate samples (10 μl at 1% [wt/vol]) of the P39a polymer were dissolved in 500 μl of 2 M trifluroacetic acid and hydrolysed at 100 °C for 4 h, as previously described (Gutierrez et al. 2008). The samples were then prepared for analysis by high-performance anion exchange chromatography using a Dionex Carbopac PA-20 column on a Dionex ICS-3000 Ion Chromatography System (Dionex Corp. Sunnyvale, USA) and eluted with 0.01 M NaOH at a flow rate of 0.3 ml/min for 20 min to elute neutral sugars and then for a further 20 min with 1 M NaOAc in 0.15 M NaOH to elute uronic acid residues. The monosaccharide composition was then quantified using external standards. The total carbohydrate content was calculated from the individual amounts of monosaccharides.

For determination of amino acid composition, acid hydrolysis was performed on 3 mg of the P39a polymer. Samples were hydrolyzed at 110 °C in 2 ml of 6 M HCl for 24 h under vacuum and then dehydrated and diluted in 0.1 M HCl. Analysis was performed using a Waters 2695 Separations Module, a 2487 Dual Absorbance Detector and a 1515 Isocratic high-performance liquid chromatography (HPLC) Pump equipped with a 300 × 3.5 mm Laborsevice 7-μm resin cation exchange column. Quantification was performed using a Sigma Amino Acid Standard (AAS18) external calibrant. The total protein content was calculated from the individual amounts of amino acids.

For molecular weight and polydispersity determination of polymer P39a, size-exclusion chromatography coupled to multi-angle laser light scattering (SEC-MALLS) was used. For this, the polymer was dissolved in distilled water at ~ 0.3% (wt/vol) and then analysed by size-exclusion chromatography at ambient temperature on a PL Aquagel guard column (Polymer Labs, Amherst, USA) which was linked in series with PL Aquagel-OH 60, PL Aquagel-OH 50 and PL Aquagel-OH 40, and was eluted with 0.1 M NaNO_3_ at a flow rate of 0.7 ml/min. The eluent was then detected online firstly by a DAWN EOS light scattering detector (Wyatt Technology, Santa Barbara, USA) and by a rEX differential refractometer (Wyatt Technology, Santa Barbara, USA). The refractive index increment, *dn/dc* was taken to be that of a typical polysaccharide (0.150 ml/g) (Harding et al. 1991; Theisen et al. 2000). Samples were run in triplicate.

For ^1^H nuclear magnetic resonance (NMR) analysis, the P39a polymer was dissolved in D_2_O (to ~ 0.7 ml) containing 1 μl of 2% acetone in D_2_O as an internal reference. Proton NMR spectra were acquired at 60 °C using a Bruker AVIII 400 MHz spectrometer. Temperature regulation utilized a BVT3200 temperature control unit. One-dimensional spectra were acquired using the Bruker pulse program ‘zgesp’ featuring a water-suppression sequence. The number of scans was set at 256, the acquisition time was ca. 1 s and a line-broadening factor of 1 Hz was imposed on the data prior to processing. TOCSY spectra were acquired using the Bruker pulse program ‘dipsi2esgpph’ featuring a water-suppression sequence. TD(1) was set at 256 W and the data was truncated in f_2_ with TD_eff_ set to 800 W, TD(2) being set to 2048 W. The mixing time was set to 120 ms.

### Effect of the exopolymer on the biodegradation of hydrocarbons

Phenanthrene was selected as a model PAH to investigate the influence of the P39a exopolymer produced by *Halomonas* strain MCTG39a on the bioavailability and biodegradation of this compound by the marine PAH-degrading ‘specialist’ *Polycyclovorans algicola* TG408 (Gutierrez et al. 2013a). For these experiments, sterile 40-ml screw-cap EPA glass vials were prepared, each containing increasing concentrations of P39a from strain MCTG39a—0.0, 0.1, 0.2 and 0.4 mg/ml—in 5 ml of ONR7a medium. Each vial was inoculated with 200 μl of cell suspension. In order to maintain a consistent mass transfer of phenanthrene in each vial, a two-phase system employing heptamethylnonane (HMN), as previously described (Gutierrez et al. 2013b), was used within which phenanthrene (PHE) was dissolved. HMN is generally considered recalcitrant to biodegradation (Wodzinski and Larocca 1977), although some studies have reported its partial breakdown by microorganisms (Katsivela et al. 2003; Rontani and Giusti 1986). Preliminary experiments in our laboratory with *P. algicola* TG408 yielded no growth on HMN as a sole carbon and energy source. Hence, it was applied in this study as a delivery system for transferring phenanthrene into the aqueous phase. For this, 2 ml of the HMN-PHE solution was carefully dispensed above the aqueous phase in each vial in order to maintain the surface area of the oil-water interface constant and standardized between treatments. All the vials were incubated with gentle rotary shaking (100 rpm) at 21 °C for a period of 12 days. Each treatment was performed in triplicate. Sampling was performed by taking 2.5-μl volumes from the HMN-PHE phase in each vial at the time of inoculation and thereafter as indicated. The samples were immediately dissolved in an appropriate volume of ethyl acetate and their absorbance measured spectrophotometrically at 251 nm. The *A*_251_ values were converted to concentrations of phenanthrene using 63,000 l mol^−1^ cm^−1^ as the extinction coefficient (Thomas and Burgess 2007). A decrease in the concentration of phenanthrene was attributed to its degradation in the aqueous phase. Incubations prepared as above, but uninoculated, were also run in order to test the potential of the P39a polymer to transfer the phenanthrene from the HMN layer into the lower aqueous phase. The purified P39a exopolymer did not serve as a source of carbon and energy since control experiments inoculated with only *P. algicola* TG408 and the P39a exopolymer did not yield any significant growth (results not shown).

Following from these experiments with a pure hydrocarbon substrate, we subsequently evaluated the P39a exopolymer for its potential to increase the bioavailability and biodegradation of a common petrochemical fuel, in this case diesel. For this, we selected a marine bacterial strain, *Halomonas* sp. strain TGOS-10, as this organism has been shown to positively respond to petrochemical contamination in the marine environment—this strain was isolated from an oil-slick seawater sample collected during the active phase of the Deepwater Horizon oil spill in the Gulf of Mexico and, based on a community sequencing survey of the impacted site, the strain had become enriched during the spill (Gutierrez et al. 2013b). For these experiments, sterile 250-ml screw-cap Erlenmeyer flasks were prepared, each containing 125 ml of ONR7a medium, 1 ml of diesel, and either 1 mg or 4 mg of the P39a exopolymer—respectively 8 mg/L and 32 mg/L final concentrations. Each flask was inoculated with 200 μl of pre-washed cells of strain MCTG39a. All the flasks were incubated with gentle rotary shaking (100 rpm) at 21 °C for a period of 7 days. Controls included flasks that were inoculated and amended with diesel in the same way but without any added exopolymer, and flasks inoculated and treated with 2 ml of 10 M HCl to act as acid-killed controls. Each treatment was performed in triplicate. To analyse for the biodegradation of the diesel, at day 7, each flask was extracted several times with 30-ml aliquots of ethyl acetate. The ethyl acetate fractions from each flask were pooled, dehydrated with anhydrous MgSO_4_, filtered and then vacuum evaporated to remove the ethyl acetate. The resultant diesel residue from each flask was then weighed. Biodegradation of the diesel by strain TGOS-10 was determined by comparing the residual weight of the diesel from incubations containing live cells of this organism to incubations with acid-killed cells.

### Statistical analysis

A Student’s *t* test was performed to test for significant differences (*P* < 0.05) in the biodegradation tests and emulsification of the different hydrocarbon substrates tested.

### Nucleotide sequence accession number

The 16S rRNA gene sequence of strain MCTG39a was deposited with GenBank under accession number MH463546.

## Results

### Isolation and identification of strain MCTG39a

Screening of a number of isolates for production of surface-active agents identified one isolate, strain MCTG39a, which was found to produce an extracellular emulsifying agent (designated P39a) with the ability to produce stable oil-in-water and water-in-oil emulsions with *n*-hexadecane and various other oil substrates. Strain MCTG39a grew well in ZM/1 medium and cell-free extracts produced high emulsification activities (EI_24_ of 100–120%) when ZM/1 was supplemented with 1% (w/v) glucose. The same cell extract only partially reduced the surface tension of the culture medium to values of between 55.0 and 60.0 mN/m during growth.

**Fig. 1:**
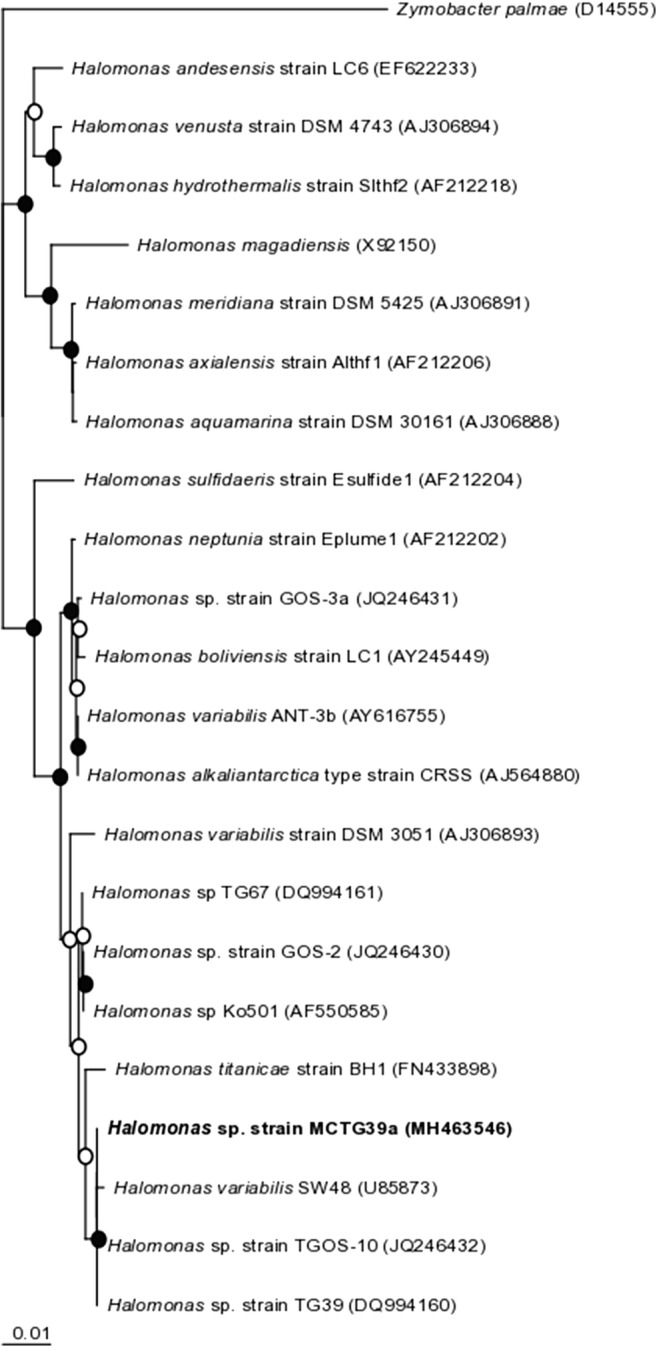
Phylogenetic tree of *Halomonas* sp. strain MCTG39a (bold) shown alongside closely related sequences and type strains from GenBank. The tree was constructed using the neighbour-joining algorithm. Filled circles indicate nodes with bootstrap values (1000 bootstrap replications) greater than 90%; open circles indicate bootstrap values greater than 60%. Accession numbers of all sequences are given in parentheses. *Zymobacter palmae* (D14555) was used as outgroup. The scale bar indicates the difference of number of substitutions per site

An almost complete sequence of the 16S rRNA gene (1439 bp) was obtained for strain MCTG39a, which was identified to be a member of the genus *Halomonas*. From a BLASTN analysis, strain MCTG39a shared 100% 16S rRNA sequence identity with *Halomonas* sp. strain TG39 (Gutierrez et al. 2007) and *Halomonas* sp. strain TGOS-10 (Gutierrez et al. 2013b)—both of which are also producers of surface-active exopolymers (Gutierrez et al. 2007, 2013b). The closest match to a type strain was with *Halomonas titanicae* BH1^T^ (99% sequence identity) isolated from the RMS *Titanic* (Sanchez-Porro et al. 2010). These and other related sequences were used to construct a neighbour-joining tree (Fig. 1). The new isolate, identified as *Halomonas* sp. strain MCTG39a, was deposited in the Deutsche Sammlung von Mikroorganismen und Zellkulturen (DSMZ) culture collection (DSM 7827).

### Cell growth and P39a production dynamics

**Fig. 2:**
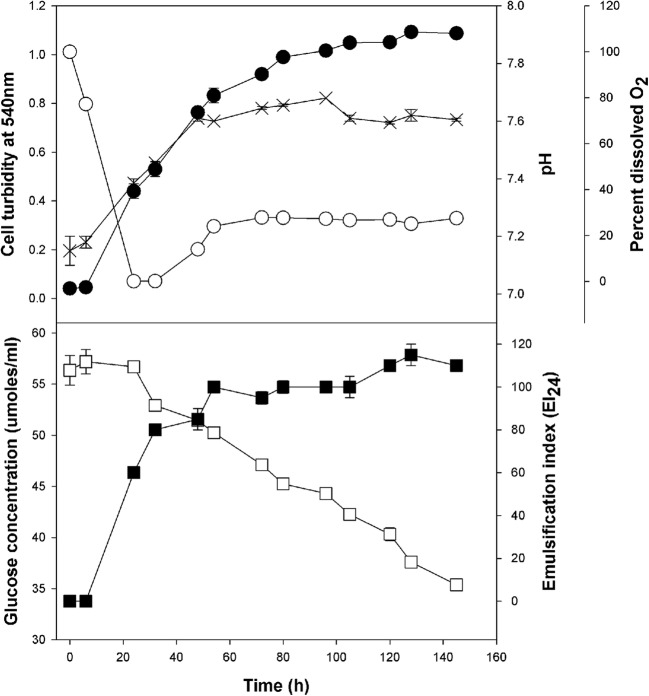
Emulsifier production during fermentation with *Halomonas* sp. strain MCTG39a in ZM/1 liquid medium amended with 1% (w/v) glucose. Emulsifying activities were derived from cell-free culture broth after the removal of cells by centrifugation. Each data point is the mean of results from triplicate samples. Solid circles, optical density at 540 nm; X, pH; empty circles, dissolved oxygen; closed squares, emulsification index after 24 h (EI_24_); empty squares, glucose concentration in μmoles mL^−1^. Some error bars are smaller than the symbols

Figure 2 shows the average dissolved oxygen, pH, turbidity (at 540 nm), glucose concentrations and emulsification index (after 24 h) over the course of batch fermentation for 142 h at a constant temperature of 28 °C. After an initial lag of ~ 21 h, glucose concentrations decreased at a steady rate of 4.1 μmoles/ml/day, and reaching lowest values by the termination of the fermentation (35 μmoles/ml). This coincided with an increase in cell turbidity (a proxy for cell growth) that reached stationary phase after around 80 h. Glucose consumption was indicated by a concomitant decrease in oxygen concentrations that stabilized by ~ 50 h to ~ 29% of oxygen saturation in the medium. This also coincided with stabilization of the pH, which increased from an initial value of ~ 7.2 to ~ 7.6 after ~ 50 h. Monitoring EI_24_ values indicated that the emulsifying exopolymer (P39a) was produced by the cells within 12 h and reached maximal levels by 120 h, although > 80% of emulsifier production was reached by 60 h. Thus, the emulsifying activity was found tightly coupled to the exponential growth of the cells.

### Chemical composition and molecular mass of the P39a exopolymer

A monosaccharide analysis of the P39a exopolymer produced by *Halomonas* sp. strain MCTG39a showed that it contained a carbohydrate content of 17.2 ± 1.9% of the total weight of dried polymer (Table 1). The polymer was composed of hexoses (rhamnose, galactose, glucose, mannose), amino sugars (glucosamine) and the uronic acid galacturonic acid. Rhamnose (35.3% ± 2.5%), galactose (19.8% ± 1.4%), glucose (16.4% ± 1.3%) and galacturonic acid (14.3% ± 1.5%) were the most abundant. All other monosaccharides of the P39a exopolymer were each present at less than 10% and together contributed about 11.5% ± 0.8% to the total carbohydrate content, with trace quantities of fucose and glucuronic acid detected. The total uronic acid content of the P39a exopolymer was 14.3%, as contributed solely by galacturonic acid.

**Table 1:** Monosaccharide composition of the P39a exopolymer produced by *Halomonas* sp. strain MCTG39a

Component	Mean mol% composition^a^
Rhamnose	35.3 ± 2.5
Fucose	Trace
Galactose	19.8 ± 1.4
Galactosamine^b^	ND
Glucose	16.4 ± 1.3
Glucosamine^b^	5.1 ± 1.2
Mannose	6.4 ± 1.4
Xylose	ND
Galacturonic acid	14.3 ± 1.5
Glucuronic acid	Trace
Total (%):^c^	17.2 ± 1.9

^a^Values are the mean of triplicate samples ± standard deviation; ND, not detected

^b^N-Acetylgalactosamine and N-acetylglucosamine are de-N-acetylated during the acid hydrolysis and are detected as galactosamine and glucosamine

^c^Total % values are expressed as the mean percentage of total dry weight of the polymer from triplicate determinations

The total amino acid content of the P39a exopolymer was 36.4% ± 0.4% (Table 2) of the total weight of dried polymer. Amino acid analysis of hydrolyzed samples identified the presence of four major amino acids—asparagine, glutamine, glycine and alanine—that combined contributed 46.9% ± 0.7% to the total amino acid content. The percent contribution of hydrophobic nonpolar amino acids to the total amino acid content was 48.0%, whereas that of polar amino acids was 44.3%. Lipid analysis did not reveal any fatty acids.

**Table 2:** Amino acid composition of the P39a exopolymer produced by *Halomonas* sp. strain MCTG39a

Component	Mean mol% composition^a^
Asparagine	12.4 ± 0.1
Threonine	6.7 ± 0.4
Serine	5.9 ± 0.3
Glutamine	14.6 ± 0.2
Proline	4.8 ± 0.5
Glycine	10.3 ± 0.1
Alanine	9.6 ± 0.6
Cystine	ND
Valine	6.6 ± 0.2
Methionine	2.0 ± 0.1
Isoleucine	4.3 ± 0.0
Leucine	7.1 ± 0.6
Tyrosine	2.3 ± 0.2
Phenylalanine	3.3 ± 0.1
Histidine	2.4 ± 0.3
Lysine	3.3 ± 0.1
Arginine	4.4 ± 0.2
Total (%)^b^	36.4 ± 0.4

^a^Values are the mean of triplicate samples ± standard deviation; ND, not detected

^b^Total % values are expressed as the mean percentage of total dry weight of the polymer from triplicate determinations

Analysis of the P39a exopolymer produced by *Halomonas* sp. strain MCTG39a by SEC-MALLS showed it composed of an average molar mass (*M*_*w*_) of 261,000 ± 12,000 g/mol, with a peak-average molar mass (*M*_*p*_) of 40,000 ± 3000 g/mol. The polydispersity index (*M*_*w*_/*M*_*n*_) of the polymer was 3.53 ± 0.13; generally, a polydispersity index of ≥ 1.6 is indicative of a polydisperse polymer (Harding et al. 1991).

**Fig. 3:**
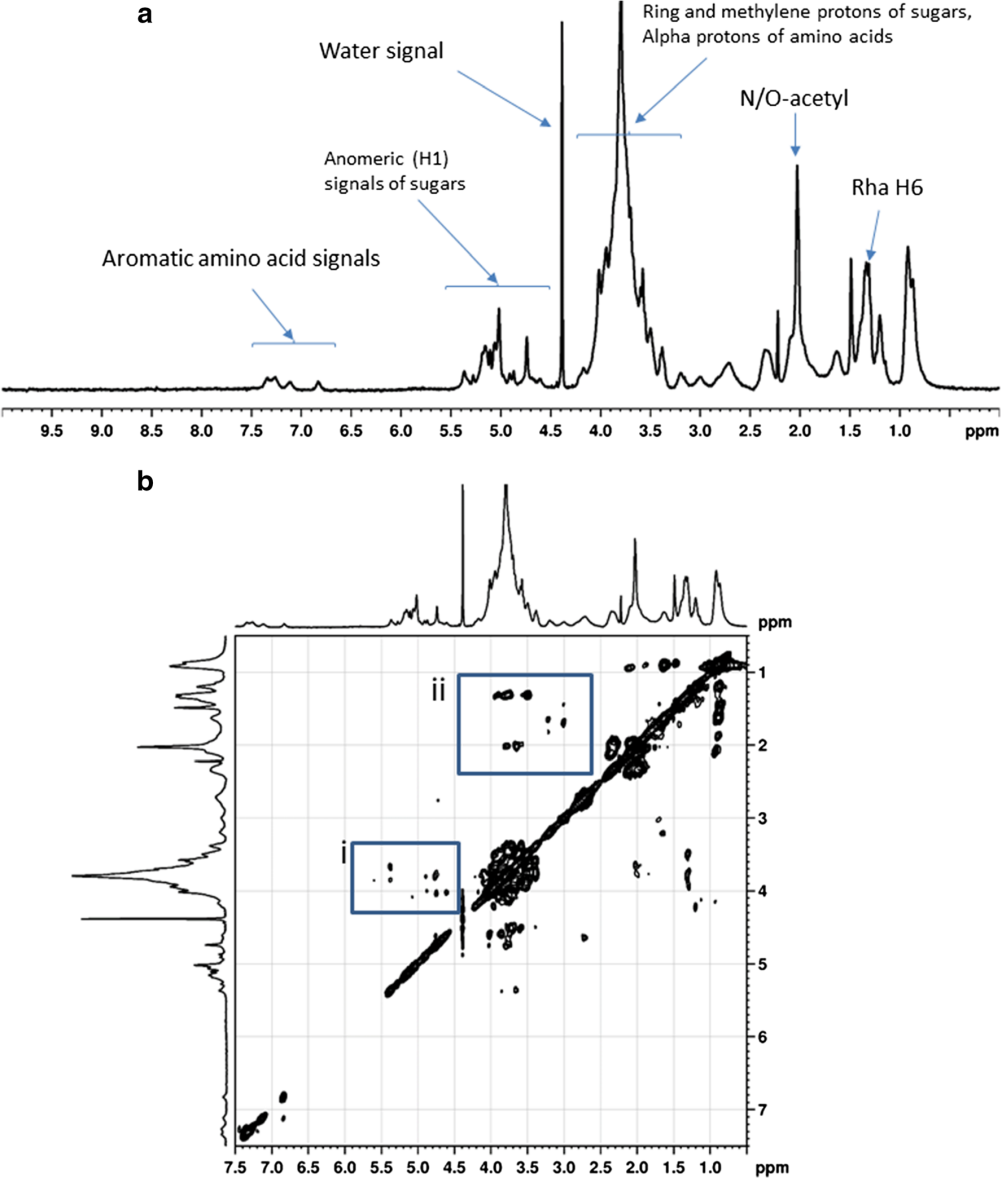
**a** Expansion 10.0–0.0 ppm of the ^1^H NMR spectrum (400 MHz, 60 °C in D_2_O) of the P39a exopolymer produced by *Halomonas* sp. strain MCTG39a. The residual partially deuterated water signal (HOD) is at about 4.8 ppm; **b** Expansion of the TOCSY spectrum of the same polysaccharide. The blue boxes indicate (i) cross-peaks linking anomeric proton signals between 4.5 and 5.5 ppm to ring and methylene proton signals between 3.5 and 4.3 ppm, and (ii) cross-peaks linking signals in the range 1.0–2.2 ppm with signals in the range 2.2 to 4.0 ppm, from aliphatic amino acid residues

Figure 3a displays the expansion of the 1D ^1^H NMR spectrum of the P39a exopolymer reflecting the peptide components of the preparation in addition to a complex carbohydrate spectrum. There is a downfield group of anomeric signals between 4.7 and 5.8 ppm, connected by TOCSY cross-peaks (Fig. 3b; box i) to an envelope of overlapping signals between 3.8 and 4.6 ppm, originating from the ring and methylene protons of sugars, in addition to α-protons of amino acids. A strong group of signals at about 1.2 ppm may arise from protons at rhamnose C6, in addition to the alanine methyl group; a peak near 2.4 ppm may include the O-acetyl methyl signal. Other prominent signals in the region between 1.0 and 3.0 ppm, with cross-peaks to the α-proton region (Fig. 3b; box ii), reflect the predominance of aliphatic amino acids in the preparation, though the presence of signals between 7.0 and 8.0 ppm indicates a minor proportion of aromatic amino acids.

### Emulsification of hydrocarbons and complex oils by the P39a exopolymer

**Fig. 4:**
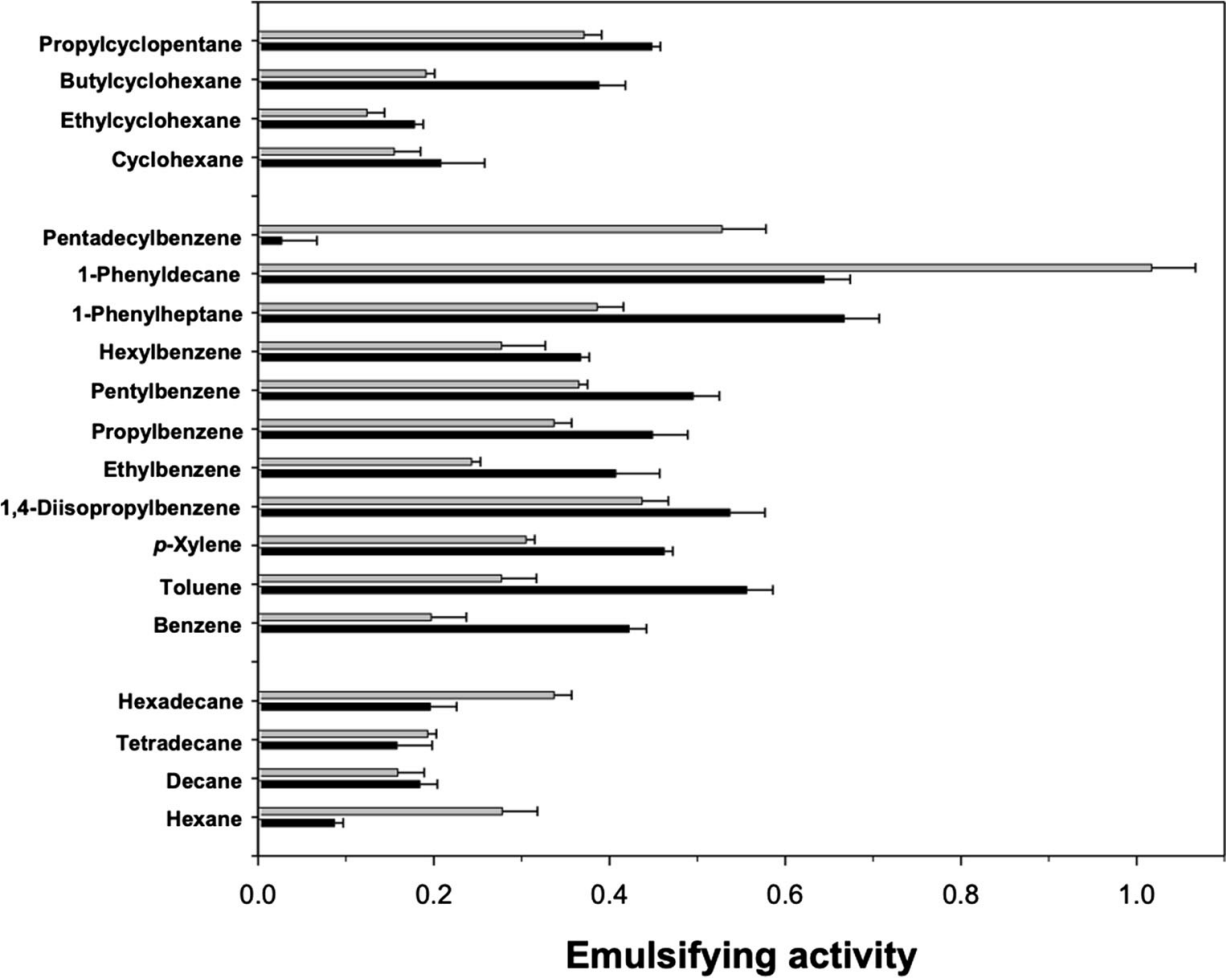
Emulsification of various aliphatic, aromatic and cycloparaffin hydrocarbons by the P39a exopolymer. Emulsification was performed in 0.1 M phosphate-buffered saline (black bars) or filtered synthetic seawater (grey bars). Emulsification activity of the lower aqueous phase was measured spectrophotometrically at 540 nm after allowing the emulsion formed to stand for 10 min, and represents a measure of oil-in-water (O/W) emulsion formation

**Fig. 5:**
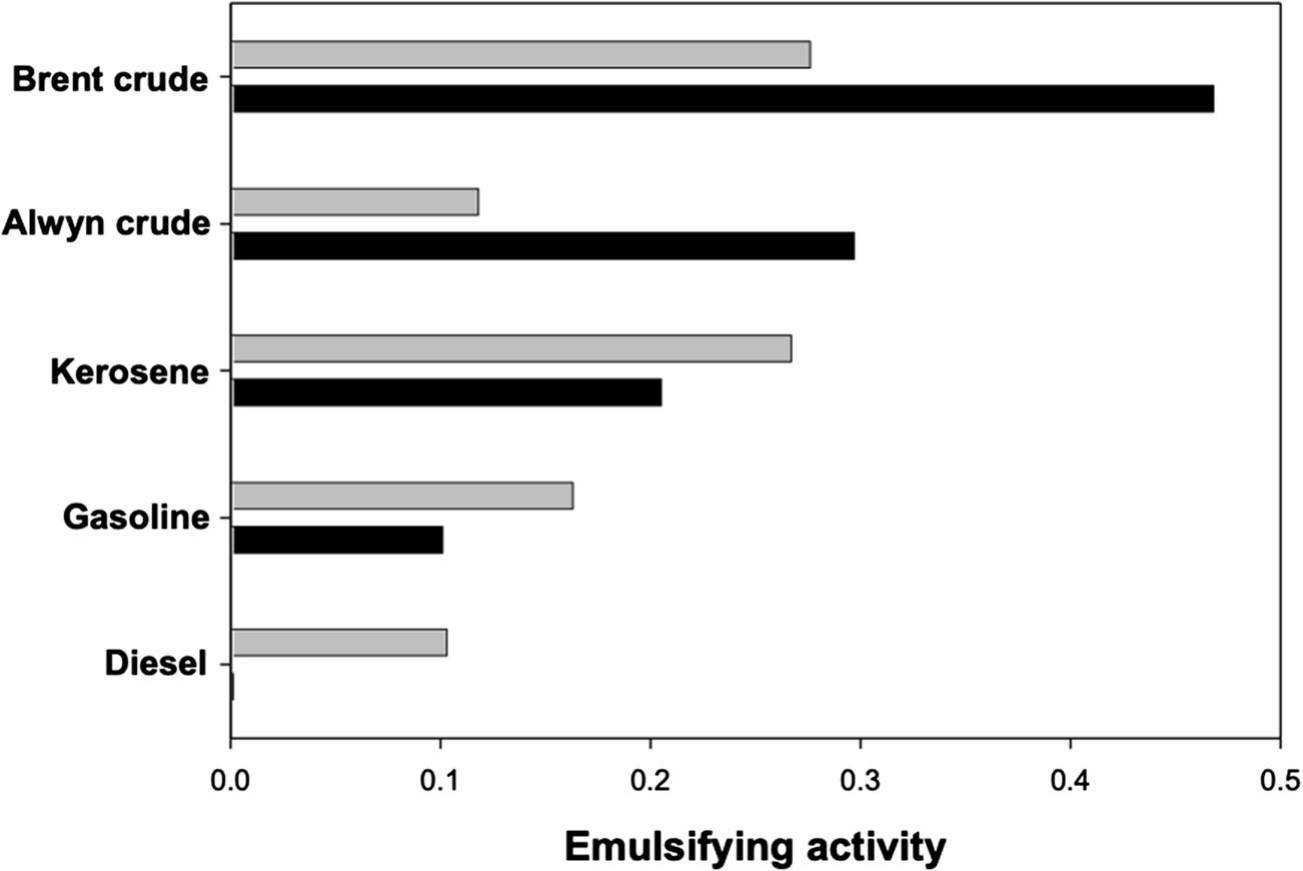
Emulsification of some crude oils and their refined derivatives by the P39a exopolymer. Emulsification was performed in 0.1 M phosphate-buffered saline (black bars) or filtered synthetic seawater (grey bars). Emulsification activity of the lower aqueous phase was measured spectrophotometrically at 540 nm after allowing the emulsion formed to stand for 10 min, and represents a measure of oil-in-water (O/W) emulsion formation

As shown in Fig. 4, the P39a exopolymer emulsified a range of straight-chain aliphatics, mono- and alkyl-aromatics and cycloparaffins. With a few exceptions, higher emulsification values were measured in 0.1 M PBS than in FSW, indicating that low ionic strength conditions, and/or possibly a lower pH, are more favourable for emulsification. Conversely, high ionic strength conditions mitigated the polymer’s emulsifying capacity. However, we measured higher emulsification values for hexane, hexadecane, 1-phenyldecane and pentadecylbenzene in FSW compared to that in 0.1 M PBS. Emulsions formed with aromatic hydrocarbons (e.g. 2-methylnaphthalene) were quite stable, and emulsification with the refined fossil fuels kerosene, gasoline and diesel also yielded higher emulsifying activities in FSW (Fig. 5). The crude oils Brent and Alwyn, which are respectively heavy and light types of oils, were highly emulsified by the P39a exopolymer, though this was more effective under lower ionic strength conditions.

### Effect of the P39a exopolymer on phenanthrene and diesel biodegradation

**Fig. 6:**
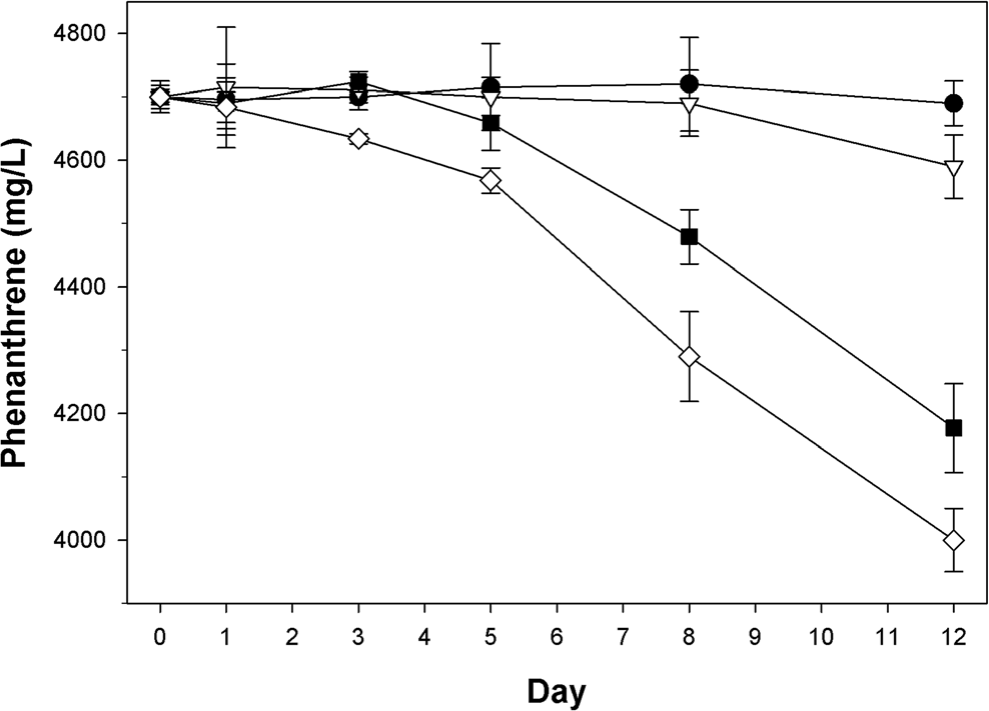
Effect of the P39a exopolymer on the degradation of phenanthrene by *Polycyclovorans algicola* strain TG408. Each data point is the mean of results from triplicate samples and represents the residual concentration of phenanthrene. Concentrations of the exopolymer tested were 0 mg/ml (solid circles), 0.1 mg/ml (inverted triangles), 0.2 mg/ml (solid squares) and 0.4 mg/ml (diamonds). Some error bars are smaller than the symbols

Figure 6 shows the polymer’s effect on the degradation of phenanthrene by the hydrocarbon-degrader *P. algicola* strain TG408. Degradation followed first-order kinetics in all four treatments with an initial lag of 8, 5, 3 and 0 days in the treatments containing, respectively, 0.0, 0.1, 0.2 and 0.4 mg/ml of the P39a exopolymer. During the time interval between day 5 and 12, the highest rates of phenanthrene degradation were measured for the treatments with 0.2 mg/ml and 0.4 mg/ml of polymer compared to that in treatments with 0.1 mg/ml or with no added polymer (Table 3). Furthermore, the degradation rates for the two treatments with the higher concentrations of the polymer (0.2 and 0.4 mg/ml) were statistically similar (65.7–80.6 mg/l/day; *P* > 0.05), though significantly higher (*P* < 0.05) compared to that of the 0.1 mg/ml treatment (15.2 mg/l/day) and untreated control (3.6 mg/l/day). More of the phenanthrene was degraded by strain TG408 in incubations with higher concentrations of the P39a polymer, with up to 14.9 ± 1.5%, 11.1 ± 2.0% and 2.3 ± 1.5% degraded in incubations amended with, respectively, 0.4 mg/ml, 0.2 mg/ml, 0.1 mg/ml of the polymer compared to only 0.2 ± 1.4% degraded in the untreated control. Incubations that were run in parallel showed that strain TG408 did not show measurable growth on the polymer as a carbon source, and no significant transfer of the phenanthrene into the lower aqueous layer was measured in uninoculated incubations containing only the P39a polymer (results not shown).

**Table 3:** Rates and extent to which phenanthrene was degraded during incubation of *P. algicola* strain TG408^T^ on increasing concentrations of the P39a exopolymer

Incubations using *P. algicola* TG408			
EPS (mg/ml)	Lag (days)	Rate at 5–12 days (mg/l/day)	Extent degraded after 12 days (%)^a^
0.0	8	3.6 ± 11.2	0.2 ± 1.4
0.1	5	15.2 ± 13.2	2.3 ± 1.5
0.2	3	65.7 ± 17.7	11.1 ± 2.0
0.4	0	80.6 ± 24.3	14.9 ± 1.5

^a^Percentage degraded of total initial phenanthrene

**Fig. 7:**
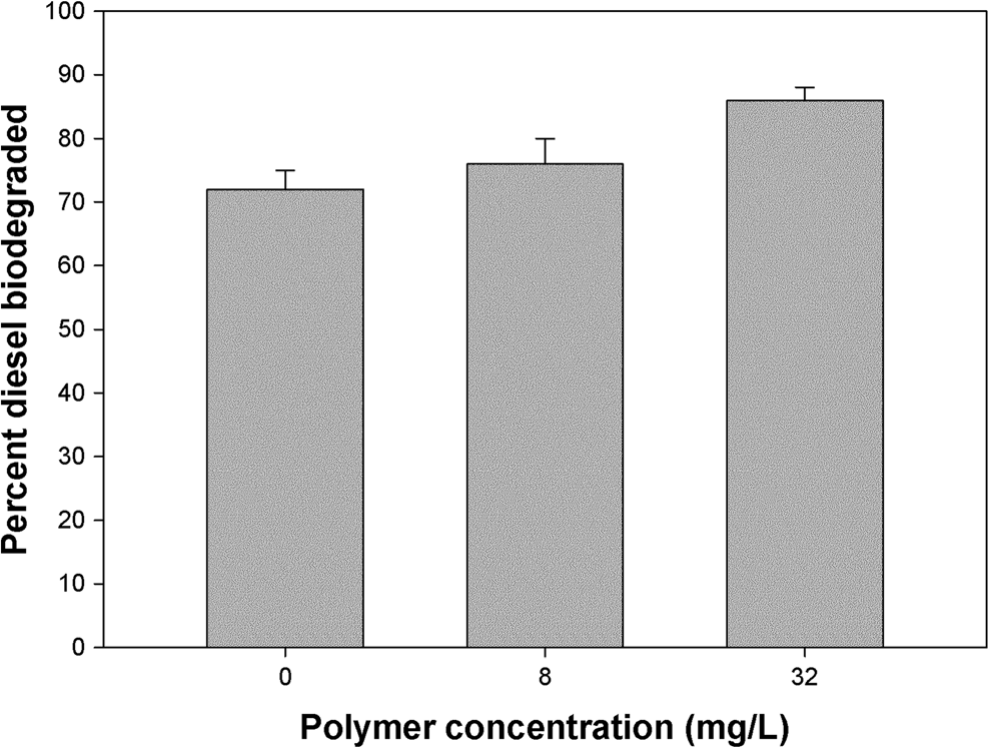
Effect of the P39a exopolymer on the degradation of diesel by *Halomonas* sp. strain TGOS-10. Concentrations of the exopolymer tested were 0, 8 and 32 mg/L. Bars represent the percentage of diesel degraded by strain TGOS-10 after a 7-day incubation

We also evaluated the effect of the P39a exopolymer on the degradation of diesel by *Halomonas* sp. strain TGOS-10 that had been isolated from a sea surface oil slick in the Gulf of Mexico during the active phase of the Deepwater Horizon oil spill (Gutierrez et al. 2013b). In the absence of any exogenously added P39a exopolymer, up to 72% of the diesel was degraded over the 7-day duration of these incubations by the strain TGOS-10 (Fig. 7). The strain exhibited the capacity to degrade the diesel, though we cannot discount that it possibly produced exopolymer during these incubations that may had facilitated this. Addition of up to 8 mg/L of the P39a exopolymer did not significantly improve on the biodegradation of the diesel. However, approximately 10% more of the diesel was biodegraded by the strain when up to 32 mg/L of the exopolymer was added.

## Discussion

Over the past two decades has seen increased interest on exopolysaccharides produced by members belonging to the genus *Halomonas* due to their rheological and/or surface-active (i.e. emulsifying or surface tension reducing) properties (Calvo et al. 1998, 2002; Martinez-Checa et al. 2002; Pepi et al. 2005). In this study, we describe the chemical and physical characterization of an extracellular water-soluble emulsifying agent, P39a, produced by a marine *Halomonas* species, strain MCTG39a.

Screening of a number of isolates for production of surface-active agents led to the identification of one isolate, strain MCTG39a, that grew on *n*-hexadecane as the sole carbon and energy source and produced a powerful extracellular emulsifying agent when grown on glucose. Bacteria that produce emulsifiers, like strain MCTG39a, are sometimes categorized based on their production of high-molecular-weight polymers, which primarily act to form stable emulsions (Rosenberg and Ron 1999). During growth in a batch fermenter using ZM/1 medium amended with glucose (1% w/v), emulsifying activity (as a proxy for production of the emulsifying agent) was tightly coupled with growth. As glucose consumption did not cease by 145 h (the termination of the fermentation), we hypothesized that production of the emulsifier could have continued should the culture have been left to proceed for longer. However, by this phase in the fermentation, we did not measure higher EI_24_ values for dilutions of the cell-free spent medium, suggesting that production of the emulsifying P39a exopolymer reached maximal yields by this point.

With the exception of rhamnose, the monosaccharide composition of the P39a exopolymer is concomitant with that of other bacterial exopolymers, which are generally rich in hexoses, like glucose and galactose (Manusco Nichols et al. 2005; Sutherland 1999). Variations in monomer composition of exopolymers can alter their properties. For example, arabinose in bacterial exopolymers aid in cell aggregation (Bahat-Samet et al. 2004), whereas deoxy sugars, like fucose and rhamnose that are found in diatom exopolymers, can mediate foaming and flocculation (Zhou et al. 1998). We note, however, that arabinose and xylose are not commonly found in bacterial exopolymers (Kenne and Lindberg 1983), and not unexpectedly they were not detected in the P39a exopolymer. Conversely, xylose was detected in an exopolymer produced by the closely related strain *Halomonas* sp. TG39, albeit in minor quantities (0.8% of total weight of dried polymer) (Gutierrez et al. 2007). The uronic acids content of the P39a exopolymer is a little below, but comparable to, the uronic acids composition (typically 20–50% of total carbohydrate content) that are generally found in exopolymers produced by marine bacteria (Kennedy and Sutherland 1987; Manusco Nichols et al. 2005). Unlike the exopolymer produced by *Halomonas* sp. TG39 which contains glucuronic acid (27.9%) as the major uronic acid (Gutierrez et al. 2007), this acid in the P39a exopolymer is present in trace quantities. Uronic acids contain an acidic carboxyl group that is ionisable at seawater pH, and which contributes a negative charge to the overall polymer (Decho and Gutierrez 2017). The carboxylate and methoxycarbonyl groups of these types of acidic sugars can mediate the adsorption of biopolymers to hydrophobic surfaces, such as oil droplets, and in turn stabilize the droplets into emulsions in aqueous media (Dea and Madden 1986; Kaplan et al. 1987; Tolstogusov 1991, 1994). The presence also of 6-deoxyhexoses (e.g. rhamnose) and increased substitution by acetyl moieties (NMR peak near 2.4 ppm) on the P39a exopolymer (see below) can also render these types of macromolecules with surface-active qualities (Dea and Madden 1986; Graber et al. 1988).

The relatively high protein content (36.4% of total polymer; Table 2) of the P39a exopolymer produced by this strain is consistent with that of other marine *Halomonas* species (Gutierrez et al. 2007, 2013b; Bèjar et al. 1998). For example, the protein content of the exopolymer produced by *Halomonas* sp. TG39 was 26.6% (Gutierrez et al. 2007), though 10% lower compared to that of the P39a exopolymer. Exopolymers produced by other halomonads have also been found to contain a protein component, and although at lower concentrations (< 10% of total polymer), it has been inferred to contribute to the emulsifying qualities of the polymers (Arias et al. 2003; Llamas et al. 2012; Quesada et al. 1993; Mata et al. 2006). Indeed, proteins can play an essential role in the emulsifying ability of some bacterial exopolysaccharides (Rosenberg et al. 1979a; Navon-Venezia et al. 1998), including for exopolymers produced by different *Halomonas* species (Mata et al. 2008; Llamas et al. 2012); we suspect the protein component of the P39a exopolymer confers a similar function, as may be effected by the ratio of total hydrophobic nonpolar to polar amino acids. This ratio was 1.1:1.0 for the P39a exopolymer, whereas it was found to be 0.6:1.0 for the exopolymer produced by the closely related strain *Halomonas* sp. TG39 (Gutierrez et al. 2007). The NMR results are consistent with a complex glycoprotein structure for the P39a exopolymer, consisting of both carbohydrate and peptide components. Interestingly, this contrasts with reports of EPS structures from other halomonads: the carbohydrate part of the sample is not a simple homopolymer such as that described for the exopolysaccharide of *Halomonas* sp. AAD6 (JCM 15723) strain (Poli et al. 2009), but a more complex compound. In addition, the monosaccharide composition of the carbohydrate is not the same as for the exopolysaccharide of *Halomonas* strain CRSS (Poli et al. 2004).

As polydispersity provides an indication of the molecular size distribution of a polymer in solution, a higher than 1.6 polydispersity index value for the P39a exopolymer denotes that it is heterogenous compared to, for example, commercially available pullulan (*I*_*p*_ ≤ 1.1). As the polymer is highly polydisperse, it is worthy to note that the weight-average is highly influenced by a small amount of very high-molecular-weight species of the exopolymer. It should be noted that the weight-average molecular weight of a mixture of components will be biased toward higher molecular weights due to the way weight-average molecular weights are calculated. This behaviour can result from the high content of anionic moieties, such as those found present on uronic acids, which are typically enriched in marine bacterial exopolymers (Decho and Gutierrez 2017).

Exopolymers from various species of *Halomonas* have been shown to emulsify hydrocarbons, crude oils and refined petroleum products (Bouchotroch et al. 2000; Calvo et al. 1998, 2002; Gutierrez et al. 2007, 2009; Martinez-Checa et al. 2002; Mata et al. 2006; Pepi et al. 2005). However, for the marine environment, there remains a paucity of knowledge pertaining to the importance of EPS produced by members of this genus and other EPS-producing bacteria to influencing the degradation of hydrocarbons. Here, we evaluated various hydrocarbons and complex oils as substrates for emulsification by the P39a exopolymer under low (0.1 M PBS) and high (FSW) ionic strength conditions, and show that high ionic strength conditions mitigated the polymer’s emulsifying capacity. We posit that this could be attributed to the presence of higher concentration of cations in reducing the polymer’s anionic charge. Our previous work with another *Halomonas* polymer showed how high ionic strength conditions can effectively reduce its cation-binding potential (Gutierrez et al. 2009). We posit that cationic species in FSW (e.g. Na^+^) would be able to neutralize anionic groups, such as sulphate and carboxyl residues, of the polymer and render it less capable of emulsifying hydrocarbon substrates (Gutierrez et al. 2009).

However, we measured higher emulsification values for hexane, hexadecane, 1-phenyldecane and pentadecylbenzene in FSW compared to that in 0.1 M PBS, suggesting that, at least for some hydrocarbon species such as these, the protein fraction of the polymer is likely to have contributed to their emulsification. This concurs with our earlier assumption that the protein component of the polymer plays an important role in emulsification. Furthermore, we also observed that the emulsions formed with aromatic hydrocarbons (e.g. 2-methylnaphthalene) were quite stable, suggesting that the P39a exopolymer likely interacts directly with the aromatic hydrocarbons by possibly steric adsorption at the oil droplet interface. The observed high emulsifying activities in FSW with the refined fossil fuels kerosene, gasoline and diesel could possibly be explained by the inherent properties of these oils, which contain higher levels of hydrocarbon species that are more effectively emulsified by the protein fraction of the polymer. Conversely, the crude oils Brent and Alwyn, which are respectively heavy and light types of oils, were more highly emulsified by the P39a exopolymer in lower ionic strength conditions, and this differential emulsifying selectivity for hydrocarbons has been reported elsewhere for other amphiphilic bacterial polymers, such as emulsan (Rosenberg et al. 1979b). A mechanism to explain this for the P39a exopolymer, however, remains unclear to us at present. Overall, the polymer displayed higher emulsifying activities for mono- and alkyl-aromatic hydrocarbons than for straight-chain aliphatics or cycloparaffins. From an ecological standpoint, this higher apparent hydrocarbon specificity for aromatics suggests that the P39a exopolymer may interact with aromatic species, such as humic substances, in the marine environment. We suspect that this interaction could potentially increase the bioavailability of these terrestrial-derived, complex aromatic substances in the water column for microbial biodegradation.

The ability of the P39a exopolymer to enhance the biodegradation of phenanthrene by other bacteria, such as the hydrocarbonoclastic marine strain *P. algicola* TG408, demonstrates the versatility of this exopolymer to enhance the bioavailability of hydrocarbon substrates to bacteria other than the producing strain, *Halomonas* sp. MCTG39a. During these experiments, the inability of *P. algicola* TG408 to utilise the exopolymer as a carbon and energy source could be explained, at least in part, by the polymer’s uronic acids content (Anton et al. 1988; Bèjar et al. 1996), or glycosidic linkages of hexosamines (Biermann 1988), which can increase the refractory nature of marine exopolymers to biodegradation (Ogawa et al. 2001).

Many species of *Halomonas* have been shown to degrade mono-aromatic (Abdelkafi et al. 2006; Garcia et al. 2004, 2005; Hinteregger and Streichsbier 1997; Munoz et al. 2001), polycyclic aromatic (Melcher et al. 2002; Calvo et al. 2002; Martinez-Checa et al. 2002; Yang et al. 2010) and aliphatic (Mnif et al. 2009; Pepi et al. 2005; Bouchotroch et al. 2000) hydrocarbons. Here, we evaluated the effect of the P39a exopolymer on the degradation of diesel by *Halomonas* sp. strain TGOS-10 that was isolated from a sea surface oil-slick sample in the Gulf of Mexico during the Deepwater Horizon oil spill (Gutierrez et al. 2013b). Interestingly, only the higher concentrations used of exogenously added P39a exopolymer effected an enhanced degradation of diesel by the strain. Petroleum diesel oil is a complex mixture of hydrocarbons that typically contain between 8 and 21 carbon atoms per molecule. The organic composition of diesel includes *n*-alkanes, branched alkanes, saturated cycloalkanes, PAHs, alkylated PAHs and alkylbenzenes which account for 27.90%, 53.87%, 7.72%, 0.26%, 3.70% and 6.55%, respectively, to its composition (Pál et al. 1998). Based on the ability of the P39a exopolymer to effectively emulsify a range of individual hydrocarbon species (Fig. 4), the combination of de novo synthesised exopolymer by strain TGOS-10 and its exogenous supplementation when added to 32 mg/L final concentration would have likely served to bolster the strain’s biodegradation of the diesel oil.
